# Gene-resolution analysis of DNA copy number variation using oligonucleotide expression microarrays

**DOI:** 10.1186/1471-2164-8-111

**Published:** 2007-04-30

**Authors:** Herbert Auer, David L Newsom, Norma J Nowak, Kirk M McHugh, Sunita Singh, Chack-Yung Yu, Yan Yang, Gail D Wenger, Julie M Gastier-Foster, Karl Kornacker

**Affiliations:** 1 Center for Childhood Cancer, Columbus Children's Research Institute and The Ohio State University, Columbus, Ohio, USA; 2 Roswell Park Cancer Institute and University at Buffalo, New York, USA; 3 Center for Cell and Developmental Biology, Columbus Children's Research Institute and The Ohio State University, Columbus, Ohio, USA; 4 Center for Molecular and Human Genetics, Columbus Children's Research Institute and The Ohio State University, Columbus, Ohio, USA

## Abstract

**Background:**

Array-based comparative genomic hybridization (aCGH) is a high-throughput method for measuring genome-wide DNA copy number changes. Current aCGH methods have limited resolution, sensitivity and reproducibility. Microarrays for aCGH are available only for a few organisms and combination of aCGH data with expression data is cumbersome.

**Results:**

We present a novel method of using commercial oligonucleotide expression microarrays for aCGH, enabling DNA copy number measurements and expression profiles to be combined using the same platform. This method yields aCGH data from genomic DNA without complexity reduction at a median resolution of approximately 17,500 base pairs. Due to the well-defined nature of oligonucleotide probes, DNA amplification and deletion can be defined at the level of individual genes and can easily be combined with gene expression data.

**Conclusion:**

A novel method of gene resolution analysis of copy number variation (graCNV) yields high-resolution maps of DNA copy number changes and is applicable to a broad range of organisms for which commercial oligonucleotide expression microarrays are available. Due to the standardization of oligonucleotide microarrays, graCNV results can reliably be compared between laboratories and can easily be combined with gene expression data using the same platform.

## Background

Array-based comparative genomic hybridization (aCGH) allows the identification of genome-wide DNA gains and losses in cancers and genetic diseases [1-3]. An ideal aCGH platform should possess the following features: 1) It should be available to study a broad range of organisms. Unfortunately, aCGH microarrays are commercially available for human and mouse studies only, leaving out other model organisms for DNA copy number studies. 2) The aCGH platform should be commercially available worldwide to make results from different laboratories easily comparable. In-house microarrays frequently show less reproducibility than commercial products [4,5]. The Microarray Quality Control Consortium (MAQC) study highlighted once again that in-house microarrays generate a much higher coefficient of variation for expression signals compared to commercial products [6]. Comparison of results generated at independent laboratories is frequently problematic when different probes are used at different laboratories. 3) Probes should span short regions to provide detailed information on regions of copy number variation (CNV); BAC clones, used as aCGH probes [7], due to their average probe length being several ten thousand nucleotides, inherently can not measure small amplified or deleted regions. 4) The platform should provide small spacing between probes to generate high density maps of CNVs; The only commercially available BAC aCGH array available measures at a median resolution of one megabase [8]. 5) Individual measurements should provide reliable data to avoid necessity of averaging multiple measurements, resulting in decreased resolution. Long oligonucleotide arrays [8,9] and SNP microarrays [10,11] depend on averaging signals from multiple probes [9,10] to eliminate false positive measurements, resulting in decreased resolution. 6) CNV measurements should be easily correlated with expression data when the same samples are studied on the genomic and transcriptomic level. BAC clones and probes designed for SNP measurement inherently are not specifically designed to interrogate transcribed genes. Therefore, combining DNA copy number and expression data needs strong bioinformatics support [11]. 7) The analytical procedure should interrogate the entire genome; the DNA labeling protocol for SNP microarrays depends on complexity reduction, leaving out significant parts of the genome from analysis [12,13].

Here we present gene resolution analysis of copy number variation (graCNV), a method utilizing the most frequently used expression microarray platform for aCGH. For human and mouse studies, this platform provides over 50,000 measurements across the genome (U133 Plus 2.0 and 430 2.0 GeneChips respectively, both Affymetrix). Furthermore, the same technology is available for more than a dozen other organisms with comparable genome coverage. The probes are short oligonucleotides and probe sets span on average short chromosomal regions. Without complexity reduction, genomic DNA is fragmented, labeled and hybridized to these microarrays. After re-annotation of probe sets for interrogation of genomic DNA, WPP, a data analysis algorithm originally developed for expression analysis, is utilized for calculation of DNA copy number variation. Since the vast majority of aCGH data available today has been generated using BAC microarrays [14], graCNV results have been compared to results from BAC microarrays with high genome coverage [15].

## Results

### Properties of the U133 Plus 2.0 Expression Array as an aCGH tool

The U133 Plus 2.0 array provides over 54,000 probe sets interrogating the human genome and over 39,000 of the probe sets measure CNVs directly within transcribed genes (Table 1). With over 19,000 genes measured on the U133 Plus 2.0 array, the majority of the predicted 20,000 to 25,000 human genes [16] is covered. Probe sets interrogate short regions and density of probe sets is high. The 19K BAC microarray used for comparison has even higher genome coverage but probes are much longer (Table 1).

### Benefits of the WPP algorithm

**Figure 1:**
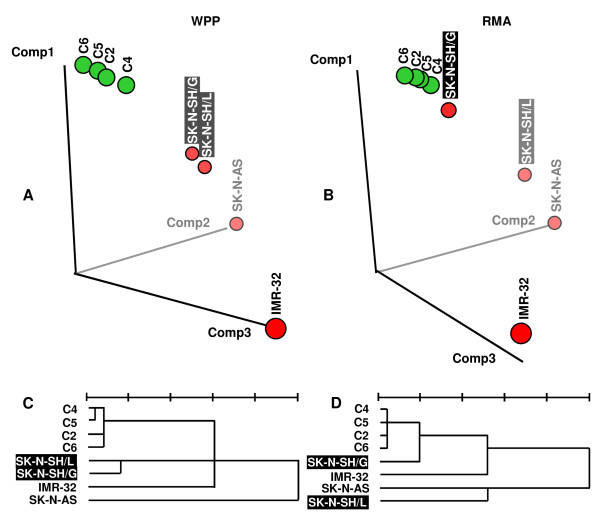
**The influence of data analysis algorithms on grouping of samples**. Principle components analysis (PCA) and hierarchical cluster analysis of DNA copy numbers of normal genomic DNAs (C2, C4, C5 and C6) and neuroblastoma cell lines (SK-N-SH/G, SK-N-SH/L, SK-N-AS and IMR-32). log2 copy number estimates calculated by WPP (left) and RMA (right) were analyzed for relative distance by PCA (top) and by hierarchical clustering (bottom). RMA separates two closely related cell lines (SK-N-SH/G and SK-N-SH/L) in PCA (Fig. 1B) and clustering (Fig. 1D), while WPP groups them together (Fig. 1A and C), clearly separated from normal genomic DNAs.

RMA, a standard data analysis algorithm for calculation of expression estimates from Affymetrix GeneChips [17] was used initially for calculation of DNA copy number differences. Principle components analysis (PCA) of RMA data grouped the normal control DNAs together but separated SK-N-SH/G and SK-N-SH/L (Fig. 1B). These two cell lines were derived from the same cell line (SK-N-SH) and have only been propagated by two different laboratories for approximately ten passages independently. Therefore, SK-N-SH/G and SK-N-SH/L should be similar to each other and should cluster together. PCA of WPP estimates grouped the normal control DNAs together, clearly separated from the cell lines and within the cell lines, SK-N-SH/G and SK-N-SH/L showed most closely related results (Fig. 1A). Hierarchical clustering of WPP estimates similarly groups related samples together (Fig. 1C), whereas RMA estimates again separated the two closely related cell lines SK-N-SH/G and SK-N-SH/L (Fig 1D). Therefore, further analysis was performed using WPP estimates of DNA copy numbers.

### Detection of copy number variations in large chromosomal regions

**Figure 2:**
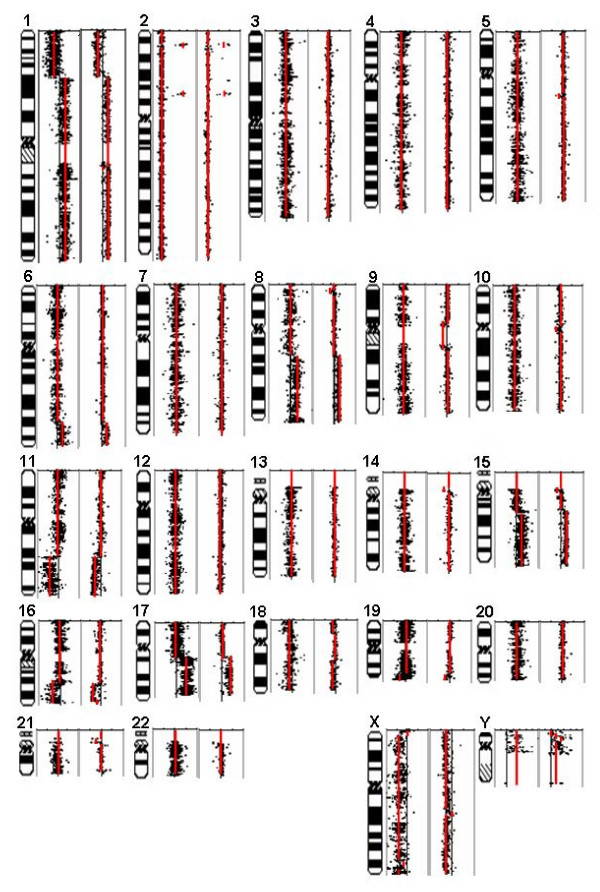
**DNA copy number variation in IMR-32 cells measured by graCNV (left) and BAC aCGH (right)**. log2 copy number differences relative to normal genomic DNA are shown on a scale from -2 to +2, except for chromosome 2 (-2 to +8). log2 copy numbers inferred from CBS are shown as red lines for segments > 1 Mb. Measurements are plotted along the physical map of chromosomes.

**Figure 3:**
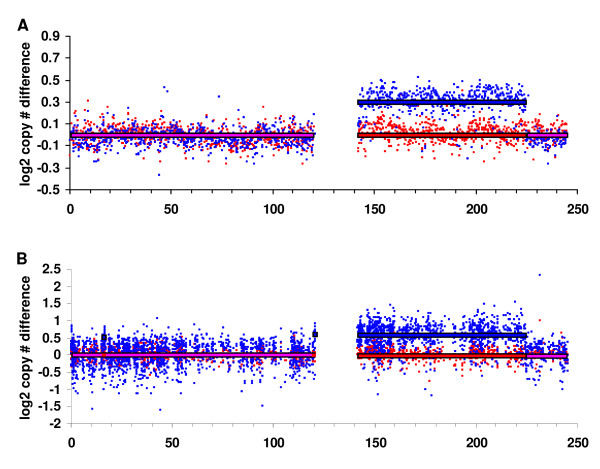
**A local copy number difference between two closely related cell lines is observed using both aCGH platforms**. SK-N-SH/G (red) and SK-N-SH/L (blue), two cell lines derived from the same patient, show copy number differences at 140 to 228 Mb of chromosome 1, where SK-N-SH/L shows gain while SK-N-SH/G shows normal DNA content. Segmentation analysis of BAC aCGH (A) and graCNV (B) highlight the same region as amplified. log2 copy numbers inferred from CBS are shown as red (SK-N-SH/G) and blue (SK-N-SH/L) lines.

For large chromosomal regions, Circular Binary Segmentation (CBS) yields similar pictures of amplification and deletion in IMR-32 neuroblastoma cells (Fig. 2) from BAC arrays and expression arrays. Two highly amplified regions on chromosome 2 (one surrounding the physical position 15 megabases (Mb) and one surrounding 68 Mb) are well known in neuroblastoma [18]. The amplicon at 15 Mb contains the MYCN oncogene and its amplification status is used for clinical sub-classification of neuroblastoma [19,20]. Deletions of 1p and 11q, as well as gains of 17q are also hallmarks of neuroblastoma [19,20] and all of these copy number variations were observed in IMR-32 cells by both platforms. The major differences observed between copy number measurements by BAC arrays and expression arrays affect centromeric regions (chromosomes 9, 10, 14, 15 and 21). These regions are highly polymorphic in normal genomic DNA[21,22]; therefore, we speculate that these differences are caused by the different normal control DNAs used for both platforms. For the remaining cell lines SK-N-AS, SK-N-SH/G and SK-N-SH/L, BAC aCGH and graCNV also provided similar pictures of copy number variations for large chromosomal regions too (Fig. 3 and data not shown). For SK-N-SH/G and SK-N-SH/L, two sub-cultures of SK-N-SH, the observed overall picture of copy number variation compared to normal DNA was very similar except for an additional amplification on 1q present in SK-N-SH/L (Fig. 3). This amplification was observed by both CGH platforms.

### High resolution analysis of copy number variations

**Figure 4:**
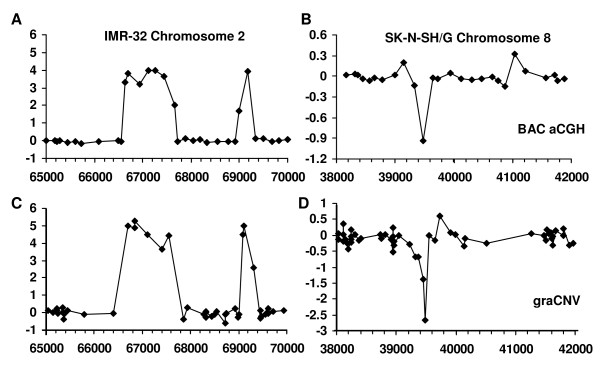
**High resolution CNV analysis using BAC aCGH and graCNV provide similar results**. In IMR-32 cells, two amplicons on chromosome 2 at 67 and 69 Mb were inferred by segmentation analysis from graCNV data (C), but only the amplicon at 67 Mb from BAC aCGH data (A). In SK-N-SH/G cells, a deletion on chromosome 8 at 39.5 Mb was inferred from graCNV data (D), but not from BAC aCGH data (B). Physical positions are shown at the x-axis in units of 1000.

At higher resolution, BAC aCGH and graCNV again provide similar pictures of amplifications and deletions in cancer cell lines. In IMR-32 cells, two recently discovered sub-amplicons at 67 and 69 Mb of chromosome two [23] have been identified by CBS in graCNV data, while only the sub-amplicon at 67 Mb was identified in BAC aCGH data (Fig. 4A and 4C). In SK-N-SH/G cells, segmentation identified from graCNV data a deletion of less than one Mb at 39.5 Mb of chromosome eight (Fig. 4B), which was not automatically detected in BAC aCGH data (Fig 4D). graCNV provides information from four independent probe sets on this deletion. BAC aCGH as used herein, with more than 50 percent genome coverage provided information from one BAC clone only. BAC aCGH provided information on two further amplicons in 2p (one at 29.6 Mb and one at 53.5 Mb) in IMR-32 cells. These regions are not covered by the U133 Plus 2.0 array (data not shown).

### Detection of copy number variations between normal genomic DNAs at a single gene locus

**Figure 5:**
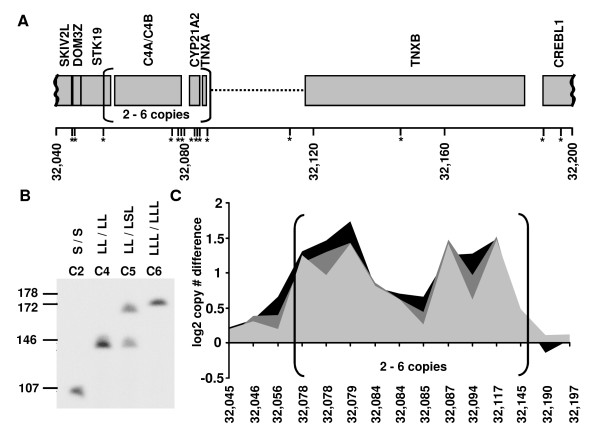
**Copy number variations at the C4/CYP21A2 locus in normal, healthy individuals**. Schematic map of chromosome 6 at 32,040 kb to 32,200 kb; the positions interrogated by probe sets are shown by asterisks; the bracket shows the region of variable copy numbers (A). Pulsed field electrophoresis analysis of the C4/CYP21A2 region of four individuals showing two copies (C2), four copies (C4), five copies (C5) and six copies (C6) of the region. The ruler to the left shows fragment size in kb; observed long and short alleles are indicated by L and S respectively (B). graCNV analysis of chromosome 6 at 32,045 kb to 32,194 kb; log2 copy number differences are shown relative to individual C2 (two copies) for individuals C4 (light gray, four copies), C5 (dark gray, five copies) C6 (black, 6 copies).

Genomic DNA of normal healthy individuals has been discovered during the last years to harbor a high number of copy number variant regions (for review see [24]). As an example, we show a region on chromosome 6, know to harbor the well-characterized CNV of the C4/CYP21A2 locus [25]. Normal, healthy individuals with 2, 4, 5 and 6 copies of the C4/CYP21A2 locus have been identified by pulsed field gel electrophoresis (Fig. 5B). This gene-level CNV is represented in the graCNV data (Fig. 5C).

### Connection of CNV and expression data

**Figure 6:**
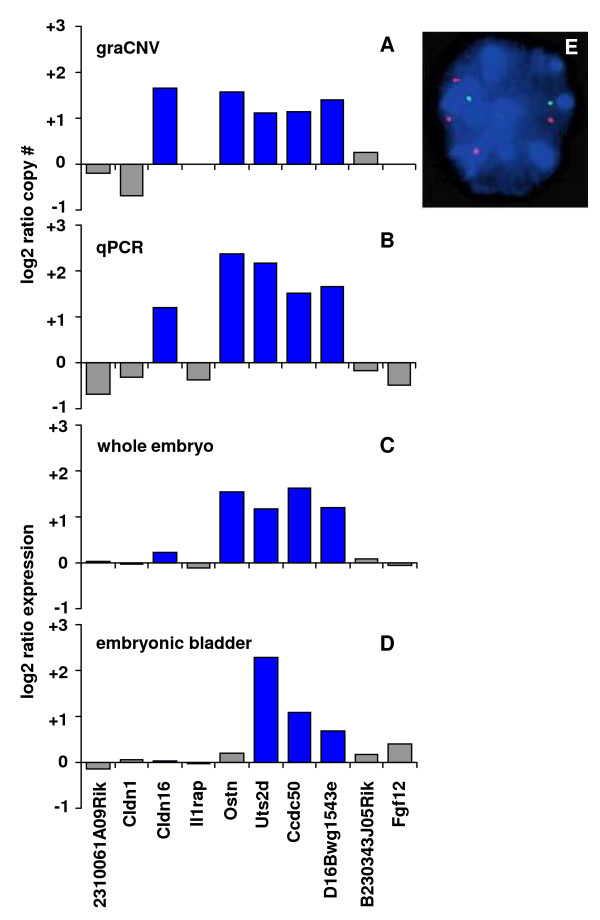
**Cohesive analysis of DNA copy number changes and differential gene expression in the mouse megabladder model**. The region of 25.9 to 28.0 Mb on mouse chromosome 16 contains 10 genes; amplification of five of these genes in homozygous mutants of the megabladder mouse was observed by graCNV (A) and confirmed by quantitative PCR (B). Differential gene expression between homozygous mutants and wild type shows overexpression of four of the amplified genes in whole embryos (C) and overexpression of three genes in the target organ, the embryonic bladder (D). (E) Interphase FISH analysis of a homozygous megabladder mutant shows two copies of chromosome 11 (green) and four copies of the duplicated region of chromosome 16 at 27.0 Mb (red).

graCNV utilizes expression arrays for CNV measurements and therefore the same microarray can be used for analysis of gene expression. Genomic and transcriptomic data can be combined easily. As an example, we show combined DNA copy number and expression data of the megabladder mouse model. An approximately 1 Mb region of chromosome 16 was duplicated in this mouse mutant (Fig. 6E), as identified by FISH analysis. graCNV showed Il1rap, a gene within the amplicon to be excluded from higher copy numbers (Fig. 6A), a finding confirmed by quantitative PCR (Fig. 6B). Expression analysis showed four of the five amplified genes to be overexpressed in mutant whole embryos (Fig. 6C), while the bladder (the affected organ) showed over-expression of three genes only (Fig. 6D).

## Discussion

Our study shows that the most frequently used commercial oligonucleotide expression microarray platform (Affymetrix) can be utilized for measurement of copy number variation. After re-annotation of probe sets, these expression arrays provide a high-resolution platform for CNV analysis.

Many aCGH platforms depend on averaging measurements from adjacent loci (moving average) to remove noise and avoid false positive reports of copy number variation [2,9,12,26]. graCNV on the other hand, reports the bias-corrected median of measurements from eleven adjacent probes within a probe set. Therefore, graCNV shows low false discovery rates (see Additional file 1) and further averaging is not vital. Affymetrix expression arrays provide for many genes more than one probe set for measurement. To allow easy interpretation of results, we averaged results of multiple probe set measuring the same gene. An even higher resolution of CNV analysis could be accomplished when the newly released expression arrays for measurement of individual exons (GeneChip Human Exon 1.0 ST arrays, Affymetrix) would be utilized.

For sample labeling, we used standard chemistry for SNP analysis from the same provider, hybridization and washing protocols are also well established by many laboratories for SNP analysis. Therefore, it should be easy to adapt graCNV by other laboratories. Affymetrix currently provides expression microarrays for over 20 organisms and graCNV can be applied to all of these organisms. Additionally, graCNV provides copy number information comparable to data generated using one of the highest density BAC aCGH arrays available.

Hybridization of genomic DNA to expression arrays produces higher background cross-hybridization (indicated by relatively high signals for mismatch probes, data not shown) than hybridization of labeled mRNA transcripts. We speculate that the reason why the RMA algorithm failed to identify the close similarity of two cell lines derived from the same donor was because RMA did not correct for sequence-specific differences in background. WPP, the algorithm introduced here for CNV measurements, utilizes mismatch probe signals for sequence-specific background correction and has been used successfully for expression analysis by one of our laboratories for several years.

Due to the fact that graCNV uses a one chip per sample principle, the range of CNVs within normal genomic DNAs can be taken into consideration for measurement of disease-related CNVs and a free software for this calculation is provided on our website[27].

## Conclusion

The present study describes a novel method of gene resolution analysis of copy number variation (graCNV) yielding high-resolution maps of DNA copy number changes and applicable to a broad range of organisms for which commercial oligonucleotide expression microarrays are available. Results are comparable to BAC aCGH with high genome coverage. Due to the standardized oligonucleotide microarrays, graCNV results can be compared between laboratories and can easily be combined with gene expression data using the same platform.

## Methods

### DNA sources

For analysis of cancer related genomic alterations, genomic DNA of neuroblastoma cell lines IMR-32, SK-N-AS and SK-N-SH (all provided by ATCC) was analyzed. SK-N-SH cells were propagated in two different laboratories for at least ten passages. The resulting cell lines were analyzed as SK-N-SH/G and SK-N-SH/L. As baseline of normal human individuals, genomic DNA from peripheral blood samples of four females (C2, C4, C5 and C6) was collected after informed consent. Genomic DNA was isolated from animals of a mouse strain (megabladder mouse), which resulted from mutagenesis during generating transgenic mice. Genetic characterization of the megabladder mouse using BAC clones containing the transgene revealed chromosome 16 at approximately 26.4 Mb to be the site of insertional mutation. FISH analysis of metaphase chromosomes further revealed this region of chromosome 16 to be translocated into chromosome 11. Therefore, wild type mice contain two copies of the genomic region surrounding 26.4 Mb on chromosome 16, heterozygous mutants contained three copies and homozygous mutants contained four copies. The megabladder mouse will be described in detail elsewhere.

### Re-annotation of expression array

Probe sets were aligned to Build 35 version of the human genome assembly by applying standalone BLAT [28] to "concatemers" formed by concatenating the non-overlapping portions of individual 25-mer probe sequences of a probe set. If BLAT did not report any match for a concatemer of a certain probe set, the probe sets was eliminated from further annotation. Homology of each alignment was computed as the percentage of concatemer bases matched and the genomic location with the highest homology was used for further annotation. The refFlat.txt.gz file[29] contains physical positions of gene locations according to the human genome assembly version Build 35 and has been used for identification of probe sets interrogating genes. When the genomic location with highest homology to a probe set overlapped with a gene in this database, the probe set was annotated to measure this particular gene. For multiple probe sets measuring the same gene, log2 copy number differences measured by individual probe sets were averaged.

### Processing of genomic DNA for graCNV using expression arrays

20 μg genomic DNA was digested using EcoRI (New England Biolabs). Fragmentation and biotin labeling using terminal transferase were performed using GeneChip Mapping 10K Xba Assay Kit (Affymetrix). Human samples were hybridized to U133plus2.0 GeneChips (Affymetrix) and mouse samples were hybridized to custom GeneChips containing 4,400 probe sets preferentially measuring genes located on chromosomes 11 and 16 (Affymetrix). Hybridization and other conditions were slightly modified from those suggested for 10K Mapping Arrays (Affymetrix) and washing conditions were carried out as suggested for 100K Mapping Arrays. A detailed description of sample processing is available in Additional file 1.

### Data analysis for expression arrays

CEL files were generated from scanned images (DAT files) using GCOS 1.4 software (Affymetrix). Probe set signals were either generated using the RMA algorithm in ArrayAssist 3.4 (Stratagene) or using the in-house developed WPP algorithm. WPP (Well behaved estimates of differential gene expression Plus probe-level p-values Plus extensible quantile scaling) software is an enhanced version of RMA [17]. WPP provides the following advanced analysis procedures which significantly increase the reliability and interpretability of calculated differentials: 1) probe-level nonparametric p-values are used to assess the statistical significance of individual calculated differentials; 2) strictly monotonic quantile scaling is used to standardize PM and MM probe intensity distributions across arrays; 3) automatic exclusion of uninformative and misinformative probes is used to increase the accuracy and precision of calculated differentials. A detailed description of the WPP algorithm is available in Additional file 1. Measurements of the four normal human DNA samples were used as baseline for measurement of copy number variation in the cell lines. CNVs of cell lines were calculated relative to the median of signals from normal samples.

### Principle components analysis and hierarchical cluster analysis

Hierarchical cluster analysis in SPSS software was applied to log2 transformed copy number estimates of probe sets using a Pearson correlation measure with furthest-neighbor distance. To exclude gender-specific differences, X-linked and Y-linked genes were excluded. Principle components analysis in SPSS software was applied with Varimax rotation to log2 transformed copy number estimates for 2,000 probe sets with the widest range of values for autosomal chromosomes.

### Construction of the Human BAC CGH array

We prepared DNA spotting solutions from sequence connected RPCI-11 BAC by ligation-mediated PCR as described previously[30]. The array contained ~19,000 BAC clones that were chosen by virtue of their STS content, end-sequence and association with cancer[15]. Each clone was spotted in duplicate on amino-silanated glass slides (Schott Nexterion typeA+) using a MicroGrid ll TAS arrayer (Apogent Discoveries). The BAC DNA products have ~80 μm diameter spots with 150 μm center-to-center spacing creating an array of ~39,000 elements. The printed slides were dried overnight and thereafter UV-crosslinked (350 mJ) in a Stratalinker 2400 (Stratagene) immediately before hybridization. A complete list of the RPCI-11 BAC clones spotted on the 19k array is available online[31].

### Labeling and Hybridization of DNA for BAC aCGH

One μg of reference and test sample genomic DNA (pooled genomic DNA of five individuals) were individually fluorescently labeled using the BioArray CGH Labeling System (Enzo Life Sciences). Initially, the DNA was denatured in the presence of the random primer at 99°C for 10 minutes in a thermalcycler, followed by a quick chill at 4°C. The tubes were transferred to ice and underwent labeling with the addition of dNTP-cyanine 3 mix (or dNTP-cyanine 5) and Klenow. Samples were incubated overnight at 37°C in a thermalcycler. The unincorporated nucleotides were removed using a QIAquick PCR purification column (Qiagen) and the labeled probe is eluted with 2 × 25 ul washes. Prior to hybridization, the test and reference probes were resuspended in 110 μl SlideHyb Buffer #3 (Ambion) containing 5 μl of 20 μg/μl Cot-1 and 5 μl of 100 μg/μl Yeast tRNA (Invitrogen), heated to 95°C for 5 minutes and placed on ice. Hybridization to the 19k CGH arrays were performed for 16 hours at 55°C using a GeneTAC hybridization station (Genomic Solutions, Inc.) as described[32]. After hybridization, the slides are automatically washed in the GeneTAC station with reducing concentrations of SSC and SDS.

### Digital Data Acquisition and Analysis for BAC aCGH

The hybridized aCGH slides were scanned using a GenePix 4200A Scanner (Molecular Devices) to generate high-resolution (5 μm) images for both Cy3 (test) and Cy5 (control) channels. Image analysis was performed using the ImaGene (version 6.0.1) software from BioDiscovery, Inc. A loess corrected log_2 _ratio of the background-subtracted test/control were calculated for each clone to compensate for non-linear raw aCGH profiles in each sample. Mapping information was added to the resulting log_2 _test/control values. The mapping data for each BAC is found by querying the human genome sequence[33] and examined for regions of large scale variation (LSV) in the human genome[8,26,34,35].

### Comparison of copy number segmentation results from expression arrays and BAC arrays

Since BAC aCGH microarray and the graCNV microarray (U133 Plus 2.0 GeneChip) have been annotated according to the human genome assembly version 35, coordinates of copy number segments were compared directly. Copy number segmentation of log2 ratios was performed in R using the DNAcopy package v1.8.1 which applies CBS (Circular Binary Segmentation) [36,37], one of the best available segmentation algorithms [38]. The undo.splits option was set to "sdundo".

### Microarray expression analysis

For expression profiling, 25 ng total RNA per sample was processed using isothermal amplification SPIA Biotin System (NuGEN Technologies, Inc.) and 2.2 μg of cDNA was hybridized per microarray. Microarrays utilized were Custom GeneChips (Affymetrix), containing probe sets to measure transcripts from mouse chromosomes 11 and 16. After 16 hours of hybridization at 45°C, washing and staining of microarrays was performed using a Fluidics Station 450 (Affymetrix); GeneChips were scanned in a GeneChip Scanner 3000 (Affymetrix). CEL files were generated from DAT files using GCOS software (Affymetrix). All steps of sample and microarray processing were performed according to manufacturer's recommendations. For calculation of differential gene expression, log2 differential expression of multiple probe sets per gene were averaged when more than one probe set was available per gene.

### Quantitative PCR

Tail samples (<1 cm) were snipped from every animal in the megabladder mouse colony. Tails were digested and DNA was isolated using Spin Doctor Genomic DNA Isolation kit (Gerard Biotech) according to the manufacturer's protocol. The DNA was resuspended in resuspension buffer included in the kit. The concentrations of the samples were determined by Nanodrop ND1000 spectrophotometer (Nanodrop), and the optical density 260/280 nm ratios were evaluated. Genomic DNA was stored at 4°C until further use. Mutant mice contain an artificial transgene in addition to the additional copies of the specified region of chromosome 16. Genotyping of mice by quantitative PCR was performed using transgene specific primers (5'-CAACCGACTCTGCATTCATCTC-3' (forward) and 5'-CTCCAGTACAGCCCTCATGTTTG-3' (reverse) and probe 5'-6FAM AAGCTTGATATCGAATTC MGBNFQ-3'. The Glucagon gene was used as internal control with primers 5'-CACAACATCTCGTGCCAGTCA-3' (forward) and 5'-ATCTGCATGCAAAGCAATATAGCT-3' (reverse), and the probe was 5'-VICT GGGATGTACAATTTCAA MGBNFQ-3'. Working concentrations of primers and probes were 18 μM and 5 μM, respectively. The multiplex PCR reactions were set up with 20 ng DNA and TaqMan Universal PCR Master Mix, No AmpErase UNG (Applied Biosystems). Reactions were performed in triplicate using the ABI series 7500 Sequence Detection System (Applied Biosystems). The initial denaturation was carried out at 50°C for 2 min, followed by 95°C for 10 min (denaturation) followed by 40 cycles of PCR reactions at 95°C for 15 sec and 60°C for 1 min. The amplification data were further analyzed using ABI 7500 System Sequence Detection Software Version 1.2.3 (Applied Biosystems). The genotype was determined by the presence of 0 versus 1 versus 2 copies of the transgene in wild type, heterozygous and homozygous mice respectively. Copy numbers of endogenous genes were determined using SYBR Green or TaqMan chemistry (both from Applied Biosystems). 10 ng of genomic DNA were used per reaction and amplification conditions for SYBR Green assays were as follows: 50°C for 2 min, 95°C for 10 min followed by 40 PCR cycles at 95°C for 15 sec, 54°C for 30 sec and 72°C for 35 sec. The data was collected at 72°C for 35 sec. TaqMan data for glucagon were used for normalization. Primers were generated for the following sequences: 2310061A09Rik (5' GCCATCTGCATATTCTTTGCTAGCA 3' forward and 5'ACATGGTTTAATGGTAGACTGGGCA 3' reverse); Cldn1 (5'CTCAACCTCCCAACTGTTAAGATGA 3' forward and 5'AACCTCTCCTATAACTGTCAGCTTC 3' reverse); Ostn (5'GAGTGTTTGCTTCAACTGTGTCAGA 3' forward and 5'AACAAGCCAGGCAGTAACTTCTTTT 3', reverse); Uts2d (5'GAGTGTTTGCTTCAACTGTGTCAGA 3' forward and 5' TAGGCTGGTAGAAGTAAACAAGCCA 3' reverse), 2610529H08Rik (5'TGGCGTCTAGGGAACTGAGTTTCTT 3' forward and 5'TGAGGAAACAGCAGTACACGATAAC 3' reverse), D16Bwg1543e (5'GCTGGCTGCAGGGAACAATCTATTT 3' forward and 5'GATGTAGACATATGAGTGGTAGTGA 3' reverse), B230343J05Rik (5'TGTGATTCATCATCGCTACAGGGAA 3' forward and 5'AACCTTCTCAAAAGCAAGGCCTTGT 3' reverse). Amplification conditions for TaqMan assays were as described above for genotyping. Commercial "Primer probe mixes" (Applied Biosystems) were used for Il1rap (ILRAP5-K1), Fgf12 (FGF12-1-A2) and Cldn16 (CLDN-I55S4).

Microarray data are deposited as GEO accession # GSE7364[39].

## Authors' contributions

HA designed the study and wrote the manuscript, DLN performed graCNV, NJN performed BAC aCGH analysis, KMM provided the megabladder model, SS performed quantitative PCR, CYY provided genetic material and information on CNVs of normal individuals, YY performed Southern blots of normal individuals, GDW performed cytogenetic research of the cell lines, JMG provided clinical information and helped writing the manuscript, KK developed data analysis algorithms and performed data analysis. All authors read and approved the final manuscript.

## Supplementary Material

Additional File 1 Supplementary Methods. The file contains two sections, a detailed description of DNA/microarray processing and of the WPP algorithm. Click here for file

## Figures and Tables

**Table 1:** Properties of the U133 Plus 2.0 expression microarray and the 19K BAC microarray as aCGH tools

**U133 Plus 2.0 microarray**			
Total probe sets	54,675	Median length of interrogated regions	535 nucleotides
Probe sets interrogating the human genome	54,045	Mean length of interrogated regions	16,703 nucleotides
Probe sets interrogating intergenic regions	14,735	Median length of regions not covered	17,447 nucleotides
Probe sets interrogating genes	39,310	Mean length of regions not covered	79,884 nucleotides
Interrogated gene regions	17,792		

**19K BAC microarray**			

Total probes	19,116 (in duplicates)	Median length of interrogated regions	167,632 nucleotides
Probes interrogating the human genome	18,411 (in duplicates)	Mean length of interrogated regions	181,473 nucleotides
		Median length of regions not covered	2,069 nucleotides
		Mean length of regions not covered	31,133 nucleotides
