# Supplementary material for: Gene-resolution analysis of DNA copy number variation using oligonucleotide expression microarrays
Source: BMC Genomics. 2007 Apr 30;8:111. doi: 10.1186/1471-2164-8-111 (PMC1868757; doi:10.1186/1471-2164-8-111)
Supplement: Additional File 1 — Supplementary Methods. The file contains two sections, a detailed description of DNA/microarray processing and of the WPP algorithm. [file 1471-2164-8-111-S1.pdf]

DNA and Microarray Processing for Microarray GCNV Analysis

STEP 1. Overnight Digestion

1. Dilute 20µg of DNA to 450µl by adding water. Add 50µl of 10x buffer and 3µl of EcoRI enzyme (5U/µl, New England BioLabs).
2. Digest overnight (16 hours) at 37ºC.

STEP 2: DNA Purification and Elution with QIAGEN MinElute 96 UF PCR Purification Plate

Reagents and Equipment
■ Manifold - QIAvac multiwell unit: QIAGEN P/N 9014579
■ MinElute 96 UF PCR Purification Kit: QIAGEN P/N 28051 (four plates), or P/N 28053 (24 plates)
■ Buffer EB (1000 ml): QIAGEN P/N120002
■ Biomek Seal and Sample Aluminum Foil Lids: Beckman P/N 538619
■ Jitterbµg 115 VAC: Boekel Scientific P/N 130000
■ Vacuum Regulator for use during the DNA clean up step. QIAGEN Vacuum Regulator (use with QIAvac manifolds): QIAGEN; P/N 19530*
* The QIAGEN protocol requires ~800 mb vacuum. If your lab does not have an internally regulated vacuum source, this vacuum regulator is strongly suggested

1. Transfer each sample into one well of the MinElute plate.
2. Apply a vacuum and maintain a ~800 mbar vacuum until the wells are completely dry. It takes about 90 minutes to dry 400 µl DNA samples.
3. Wash the DNA products by adding 50 µl molecular biology water and dry the wells completely (approximately 20 minutes). **Repeat this step 2 times** for a total number of 3 water washes.
4. Switch off vacuum source and release the vacuum.
5. Carefully remove the MinElute plate from the vacuum manifold.
6. Gently tap the MinElute plate on a stack of clean absorbent paper to remove any liquid that might remain on the bottom of the plate.
7. Add 40 µl EB buffer to each well. Cover the plate with PCR plate cover film.
8. Moderately shake the MinElute plate on a plate shaker, e.g., Jitterbug for 5 minutes.
9. Recover the purified DNA by pipetting the eluate out of each well. For easier recovery of the eluates, the plate can be held at a slight angle.

STEP 3: Quantification of Digested Genomic DNA

1. Use a Nanodrop (spectrophotometer) to determine the purified DNA yield.
2. Read the absorbance at 260 nm. Ensure that the reading is in the quantitative range of the instrument.
3. Normalize the DNA concentration to 20 µg of DNA product per 45 µl by adding EB buffer (10mM Tris-HCl, pH 8.5).
4. Transfer 45 µl (20 µg) of each of the purified DNA to corresponding wells of a new plate for fragmentation.

Pre-heat thermal cycler to 37ºC.

STEP 4: Fragmentation

Reagents and Equipment

■ Fragmentation Reagent (DNase I): Affymetrix, P/N 900131, available in Box 3 of the GeneChip® Mapping 10K Xba Assay Kit, P/N 900441
■ 10 X Fragmentation Buffer: Affymetrix, P/N 900422, available in Box 3 of the GeneChip® Mapping 10K Xba Assay Kit, P/N 900441

Examine the label of the GeneChip Fragmentation Reagent tube for U/µl definition, and calculate dilution.

Y = number of µl of stock Fragmentation Reagent
X = number of U of stock Fragmentation Reagent per µl (see label on tube)
0.048 U/µl = final concentration of diluted Fragmentation Reagent
125 µl = final volume of diluted Fragmentation Reagent*

$$Y = \frac{0.048 U/\mu l * 125 \mu l}{X U/\mu l}$$

Dilute the stock of Fragmentation Reagent to 0.048 U/µl using Fragmentation Buffer and Molecular Biology Water ON ICE and vortex at medium speed for 2 seconds.

Two examples of dilution are listed below for two different concentrations of Fragmentation Reagent.

Reagent	2 units/µl	3 units/µl
Fragmentation Reagent	3 µl	2 µl
10X Fragmentation Buffer	12.5µl	12.5 µl
H2O	109.5 µl	110.5 µl
Total	125 µl	125 µl

Fragmentation Procedure

1. Pre-heat thermal cycler to 37°C.
2. Add 5 µl 10X Fragmentation Buffer to each sample on the fragmentation plate ON ICE and vortex at medium speed for 2 seconds. Place back on ice.
3. Add 5 µl of diluted Fragmentation Reagent (0.048 U/µl) to the fragmentation plate containing Fragmentation Mix ON ICE. Pipet up and down several times to mix. The total volume for each sample is listed below.

Reagent	Volume/Sample
**Purified DNA (20 µg in EB buffer)**	**45 µl**
**10X Fragmentation Buffer**	**5 µl**
**Diluted Fragmentation Reagent (0.048U/µl)**	**5 µl**
**Total**	**55 µl**

4. Vortex the fragmentation plate at medium speed for 2 seconds, and spin briefly at 2,000 rpm for 1 minute.
5. Place the fragmentation plate in pre-heated thermal cycler (37°C)

6. Run the following program:

Temperature	Time
**37º C**	**30 Minutes**
**95º C**	**15 Minutes**
**4º C**	**Hold**

7. Spin the plate briefly after fragmentation reaction.

STEP 5: Labeling

Reagents

- GeneChip DNA Labeling Reagent: Affymetrix; P/N 900430, available in Box 3 of the GeneChip® Mapping 10K Xba Assay Kit, P/N 900441
- Terminal Deoxynucleotidyl Transferase (30 U/µl): Affymetrix; P/N 900426, available in Box 3 of the GeneChip® Mapping 10K Xba Assay Kit, P/N 900441
- 5X Terminal Deoxynucleotidyl Transferase Buffer: Affymetrix; P/N 900425, available in Box 3 of the GeneChip® Mapping 10K Xba Assay Kit, P/N 900441

Labeling Procedure

1. Prepare Labeling Mix as Master Mix ON ICE and vortex at medium speed for 2 seconds (for multiple samples, make 5% excess).

Reagent	1X	Final Conc. In Sample
5X TdT Buffer	14 µl	1X
GeneChip DNA Labeling reagent (5mM)	2 µl	0.143 mM
TdT (30U/µl)	3.4 µl	1.5 U/µl
Total	19.4 µl	

2. Aliquot 20 µl of Labeling Master Mix into the fragmentation plate containing ~54 µl of fragmented DNA samples as follows:

Reagent	Volume/Rx
Framented DNA (from Fragmentation step)	54 µl
Labeling Mix	20 µl
Total	74 µl

3. Seal the plate tightly with a plate cover.
4. Vortex the plate at medium speed for 2 seconds, and briefly spin the plate at 2,000 rpm for 1 minute.

5. Run the following program:

Temperature	Time
37º C	2 hours
95º C	15 minutes
4º C	Hold

STEP 6: Target Hybridization

Reagents

- ■ 5M TMACL (Tetramethyl Ammonium Chloride): Sigma; P/N T3411
- ■ 10% Tween-20: Pierce; P/N 28320 (Surfactamps); diluted to 3% in molecular biology grade water
- ■ MES hydrate: Sigma; P/N M5287
- ■ MES Sodium Salt: Sigma; P/N M5057
- ■ DMSO: Sigma; P/N D5879
- ■ EDTA: Ambion; P/N 9260G
- ■ Denhardt's Solution: Sigma; P/N D2532
- ■ HSDNA (Herring Sperm DNA): Promega; P/N D1815
- ■ Human Cot-1: Invitrogen; P/N 15279-011
- ■ Oligonucleotide Control Reagent: Affymetrix; P/N 900440, available in Box 3 of the GeneChip® Mapping 10K Xba Assay Kit, P/N 900441

Reagent Preparation

12 X MES Stock
(1.22 M MES, 0.89 M [Na+])

For 1000 ml:
70.4 g MES Hydrate
193.3 g MES Sodium Salt
800 ml Molecular Biology Grade water
Mix and adjust volume to 1,000 ml.

The pH should be between 6.5 and 6.7.
Filter through a 0.2 µM filter.

1. Prepare the following Hybridization Cocktail Master Mix:

Reagent	1X
MES (12X; 1.22 M)	12 µl
DMSO (100%)	26 µl
Denhardt's Solution (50X)	13 µl
EDTA (0.5 M)	3 µl
HSDNA (10mg/ml)	3 µl
Control oligonucleotide BS (3 nM)	3.7 µl
20X Eukaryotic Hybridization Controls	11 µl
Tween-20	1 µl
TMACL (5M)	140 µl
Total	212.7 µl

2. Transfer each sample from the plate into a 1.5 ml tube. Aliquot 190 µl of the Hybridization Cocktail Master Mix into the 70 µl of labeled DNA samples.
3. Heat the 260 µl of hybridization mix and labeled DNA at 95°C in a heat block for 10 minutes to denature.
4. Cool down on crushed ice for 10 seconds.
5. Spin briefly at 2,000 rpm for 1 minute in a microfuge to collect any condensate.
6. Place the tubes at 48°C for 2 minutes.
7. Inject 210 µl denatured hybridization mix into the array.
8. Hybridize at 48°C for 16 to 18 hours at 60 rpm.

7. Washing and Staining of Microarrays

Reagents and Equipment

- Water, Molecular Biology Grade, BioWhittaker Molecular Applications / Cambrex, P/N 51200
- Distilled water, Invitrogen Life Technologies, P/N 15230147
- Acetylated Bovine Serum Albumin (BSA) solution (50 mg/ml), Invitrogen Life Technologies, P/N 15561-020
- 20X SSPE (3M NaCl, 0.2M NaH_2PO_4, 0.02 M EDTA), BioWhittaker Molecular Applications / Cambrex, P/N 51214
- Anti-streptavidin antibody (goat), biotinylated, Vector Laboratories, P/N BA-0500
- R-Phycoerythrin Streptavidin, Molecular Probes, P/N S-866
- 10% surfact-Amps20 (Tween-20), Pierce Chemical, P/N 28320
- Bleach (5.25% Sodium Hypochlorite), VWR Scientific, P/N 21899-504 (or equivalent)
- Denhardt's Solution, 50X concentrate: Sigma; P/N D2532
- MES hydrate, Sigma-Aldrich, P/N M5287
- MES Sodium Salt, Sigma-Aldrich, P/N M5057
- 5M NaCl, RNase-free, DNase-free, Ambion, P/N 9760G

GeneChip® Mapping 10K 2.0 Assay Manual 57

Reagent Preparation
Wash A: Non-Stringent Wash Buffer
(6X SSPE, 0.01% Tween 20)
For 1000 ml:
300 ml of 20X SSPE
1.0 ml of 10% Tween-20
699 ml of water

Filter through a 0.2 µm filter.
Store at room temperature.

Wash B: Stringent Wash Buffer
(0.6X SSPE, 0.01% Tween 20)
For 1000 ml:
30 ml of 20X SSPE
1.0 ml of 10% Tween-20
969 ml of water
Filter through a 0.2 µm filter.
Store at room temperature.
0.5 mg/ml Anti-Streptavdin Antibody
Resuspend 0.5 mg in 1 ml of water.
Store at 4°C.
GeneChip® Mapping 10K 2.0 Assay Manual 59

12X MES Stock Buffer
(1.22M MES, 0.89M [Na+])
For 1,000 ml:
70.4g of MES hydrate
193.3g of MES Sodium Salt
800 ml of Molecular Biology Grade water
Mix and adjust volume to 1,000 ml.
The pH should be between 6.5 and 6.7.
Filter through a 0.2 µm filter.
1X Array Holding Buffer
(Final 1X concentration is 100 mM MES, 1M [Na+], 0.01% Tween-20)
For 100 ml:
8.3 ml of 12X MES Stock Buffer
18.5 ml of 5M NaCl
0.1 ml of 10% Tween-20
73.1 ml of water
Store at 2°C to 8°C, and shield from light
Do not autoclave. Store at 2°C to 8°C, and shield from light.
Discard solution if yellow.

Preparing the Staining Reagents

Prepare the following reagents. Volumes given are sufficient for one probe array. Mix well.

Stain Buffer

Components	1X	Final Concetration
H2O	666.7 µl	
SSPE (20X)	300 µl	6X
Tween-20	3.3 µl	0.01%
Dehhardts (50X)	20	1X
Subtotal	990 µl	
Subtotal/2	495	

SAPE Stain Solution:

Components	Volume	Final Concentration
Stain Buffer	495 µl	1X
1 mg/ml Straptavidin Phycoerythrin (SAPE)	5.0 µl	10ug/ml
Total	500 µl	

FS450 Users: *A vial containing SAPE Stain Solution must be placed in sample holder 1 for each module used.*
FS400 Users: *A vial containing SAPE Stain Solution must be used for the first and third stains.*
66 Probe Array Wash and Stain

Antibody Stain Solution

Components	Volume	Final Concentration
Stain Buffer	495 µl	1X
0.5 mg/ml biotinylated antibody	5 µl	5 ug/ml
Total	500 µl	

FS450 Users: *A vial containing Antibody Stain Solution must be placed in sample holder 2 for each module used.*
FS400 Users: *A vial containing Antibody Stain Solution must be used for the second stain.*

Use wash protocol: Mapping 100Kv1 for U133Plus2.0 GeneChips

F450 Users: **Be sure to place SAPE solution in holder 1, Antibody Solution in holder 2, and 1X Holding buffer in holder 3.**

WPP Algorithm

1. Well-behaved Estimator of Differential Gene Expression Plus Quantile Scaling Plus Probe-level Testing)

1.1. WPP is “well behaved” in the sense that its expression estimates can remain accurate even if some probe pairs are “badly behaved” (e.g. high outlier PM intensity, near-zero PM sensitivity, negative PM sensitivity, strong between-array variations in PM cross hybridization, etc.).

2. Bias correction

2.1 Quantile scaling (Q-scaling)

2.1.1 Exclusion of high outliers

2.1.1.1. For each array j, $1 \le j \le N$, determine the Langmuir saturation limit for PM intensities: L_j

2.1.1.2. For each j, recode all PM intensities above L_j (i.e. high outliers) as missing values.

2.1.2 Calculation of Q-scaling factors

2.1.2.1. For each j and all PM intensities below L_j, calculate a set of quantiles: Q_{ij}, $1 \le i \le M$

2.1.2.2. For each i and j, calculate the Langmuir-corrected quantile: $\hat{Q}_{ij} = \left(Q_{ij}^{-1} - L_j^{-1}\right)^{-1}$

2.1.2.3. For each i, calculate the average corrected quantile: $\overline{\hat{Q}}_{i\cdot} = N^{-1}\sum_j \hat{Q}_{ij}$

2.1.2.4. For each i and j, calculate the Q-scaling factor: $QS_{ij} = \overline{\hat{Q}}_{i\cdot} \big/ Q_{ij}$

Comments

While Langmuir correction is optional, early exclusion of high outliers is necessary for robust calculation of Q-scaling factors.

Since high intensity values are generally unique, while low values are often repeated hundreds of times, WPP fits a set of M exponentially increasing quantile frequencies, which terminate at 1.

The WPP default value of M is 1000. Small changes in M generally have little effect.

2.1.3. Calculation of Q-scaled intensities

2.1.3.1. For each i, $1 \le i < M$, array j and PM intensity x satisfying $Q_{ij} \le x \le Q_{(i+1)j}$, calculate the logarithmically interpolated Q-scaling factor:

$$LIQS(x) = QS_{ij} \text{ if } x = Q_{ij} \text{ ; else}$$

$$LIQS(x) = \exp\{\log(QS_{ij}) + \alpha(x) \cdot \log(QS_{(i+1)j} / QS_{ij})\}$$
$$\text{where } 0 < \alpha(x) = \log(x/Q_{ij}) / \log(Q_{(i+1)j} / Q_{ij}) \le 1$$

2.1.3.2. For each PM intensity x, calculate the Q-scaled PM intensity*:*

$$\tilde{x} = x \cdot LIQS(x)$$

Comment

The use of logarithmically interpolated Q-scaling factors guarantees that Q-scaling is monotonic and one-to-one, i.e. $\tilde{x}' > \tilde{x}$ if and only if $x' > x$. *In contrast, Bolstad quantile normalization generally becomes one-to-many for recurrent low-intensity values, potentially adding significant bias depending on the original ordering of the probeset data.*

2.1.4. Calculation of Q-scaled MM intensities

2.1.4.1. Apply the entire Q-scaling procedure to MM intensities

2.2. Probe-specific bias correction

2.2.1. Correction for PM background cross hybridization

2.2.1.1. For each probepair p in probeset s on array j, the Q-scaled PM and MM intensities are: $QSPM_{psj}$ and $QSMM_{psj}$ respectively.

2.2.1.2. For each probepair p in probeset s on array j and fixed β, $0 < \beta < 1$, calculate the log2 background-corrected value of $QSPM_{psj}$:

$$L2BCQSPM_{psj} = \begin{cases} \log_2(QSPM_{psj} - QSMM_{psj}) \text{ if } QSMM_{psj} < \beta \cdot QSPM_{psj} \\ \log_2((1-\beta) \cdot QSPM_{psj}) \text{ otherwise} \end{cases}$$

Comments

The constant factor $(1-\beta)$ *constrains the maximum amount of background correction while preserving transcript-specific sensitivity, even when Q-scaled MM intensities exceed the paired Q-scaled PM intensities. In contrast, MAS5 replaces PM intensities*

with constant array-specific background intensities whenever PM intensities fall below the paired MM intensities, needlessly masking any transcript-specific sensitivity.

The WPP default value of β *equals* $15/16$. *Lower values of* β *could under-correct any PM intensities that involve unusually high PM cross hybridization; higher values of* β *could over-correct any PM intensities that are paired with unusually high MM intensities. The default* β *value has been found to yield significantly more accurate expression ratios than GCRMA (see Example 2 below).*

2.2.2. Correction for PM sensitivity

2.2.2.1. For each probepair p in probeset s, the median value of $L2BCQSPM_{psj}$ over all j is:

$$L2ProbeMedian_{ps}$$

2.2.2.2. For each probeset s, the median value of $L2BCQSPM_{psj}$ over all included p and j is:

$$L2ProbesetMedian_{s}$$

2.2.2.3. For each probepair p in probeset s on array j, calculate the sensitivity-corrected value of $L2BCQSPM_{psj}$:

$$SCL2BCQSPM_{psj} = L2BCQSPM_{psj} + L2ProbesetMedian_{s} - L2ProbeMedian_{ps}$$

2.2.3. Iterative exclusion of uninformative and misinformative probes

2.2.3.1. For each probeset s on array j, the median value of $SCL2BCQSPM_{psj}$ over all included p is:

$$L2ArrayMedian_{sj}$$

2.2.3.2. For each probeset s on array j, calculate the signum of $\left(L2ArrayMedian_{sj} - L2ProbesetMedian_{s}\right)$**:**

$$\mathrm{sgn}_{sj} = \begin{cases} +1 \text{ if } L2ArrayMedian_{sj} > L2ProbesetMedian_{s} \\ -1 \text{ if } L2ArrayMedian_{sj} < L2ProbesetMedian_{s} \\ 0 \text{ otherwise} \end{cases}$$

2.2.3.3. A value of $SCL2BCQSPM_{psj}$ is regarded as directionally inconsistent if:

$$\mathrm{sgn}_{sj} \cdot \left(SCL2BCQSPM_{psj} - L2ProbesetMedian_s \right) < 0$$

2.2.3.4. For each probeset s, iteratively exclude the least informative probepairs.

2.2.3.4.1. Stop before excluding a majority of probepairs.

2.2.3.4.2. Stop if there are no directional inconsistencies.

2.2.3.4.3. Exclude the probepair that has the highest number of directional inconsistencies and the lowest signum-correlated measure of differential expression:

$$\sum_j \mathrm{sgn}_{sj} \cdot \left(SCL2BCQSPM_{psj} - L2ProbesetMedian_s \right)$$

2.2.3.4.4. Recalculate all values affected by probepair exclusion:

$L2ProbesetMedian_s$

$SCL2BCQSPM_{psj}$

$L2ArrayMedian_{sj}$

3. Calculation of robust unbiased expression estimates

3.1. Array-specific estimates

For each probeset s on array j, the array-specific log2 expression estimate is: $L2ArrayMedian_{sj}$

3.2. Group-specific estimates

For each probeset s and array-group G, calculate the pooled group-specific log2 expression estimate as the median value of $SCL2BCQSPM_{psj}$ over all included p in s and all j in G.

4. Probe-level multi-array tests for statistically significant differential expression

4.1. Calculate p-values based on the nonparametric Mood test for equal medians.

Three representative examples based on the Affymetrix HG-U133A_tag Latin Square data set

Latin Square features

The data set consists of 3 technical replicates of 14 separate hybridizations of 42 spiked transcripts in a complex human background at concentrations ranging by factors of two from 0.125pM to 512pM. Thirty of the spikes are isolated from a human cell line, four

spikes are bacterial controls and eight spikes are artificially engineered tag sequences believed to be unique in the human genome. A single background hybridization mix is used for all 42 hybridizations.

Each of the four bacterial control spikes is detected by 6 probesets, increasing the total number of spiked probesets from 42 to 62. In addition, Affymetrix identifies 3 other probesets that share probes with spiked probesets, bringing the total number of documented spiked probesets to 65.

Analysis of the data reveals that four additional probesets detect undocumented transcripts which are maximally abundant in Expt 14 and are clearly detectable in Expts 11-14, possibly due to contamination of the corresponding spike mix. The undocumented spikes are:

LCK (204890_s_at, 204891_s_at)

MGC16824 (203173_s_at)

CHI3L2 (213060_s_at)

None of the undocumented spikes are included in the results reported for the following examples.

Example 1: Calculation of p-values and error rates for the detection of spikes in Expts 7 and 8

The p-values for MAS5, RMA and GCRMA were calculated as usual by applying a t-test to the two sets of triplicate expression estimates.

It is convenient to express p-values as NLP values (Negative Log10 P-value), i.e.

$$NLP = -\log_{10}(p\_value)$$

The spiked TagD expression ratio for Expts 7 and 8 is 2:1 and provides a representative example.

The WPP estimated TagD log2 expression ratio is 0.745 with an NLP of 14.02. The extremely high NLP results from the use of a probe-level multi-array test and is certainly high enough to control the FWER (Family-Wise Error Rate). In fact, WPP associates zero false positives with this NLP; i.e. no true-negative (not spiked) probesets are assigned the same or higher NLP. Moreover, WPP associates 32 true-positive (spiked) probesets with this NLP.

The MAS5 estimated TagD log2 expression ratio is 0.975 with an NLP of 3.55. The comparatively low NLP results from the use of a probeset-level multi-array t-test and is far too low to control the FWER. In fact, MAS5 associates 18 false positives and 35 true positives with this NLP. The corresponding FDR (False Discovery Rate) is 34% and 4 of the false-positive log2 expression ratios are above 2. This MAS5 result is inadequate for reliable detection of spiked differential expression, even when all samples share the same background hybridization mix.

The RMA estimated TagD log2 expression ratio is 1.029 with an NLP of 3.37. As with MAS5, the low NLP results from the use of a probeset-level multi-array t-test and is far too low to control the FWER. In fact, RMA associates 14 false positives and 41 true positives with this NLP. The corresponding FDR is 25% and the highest false-positive log2 expression ratio is below 0.2. This RMA result is conditionally adequate for reliable detection of spiked differential expression when all samples share the same background hybridization mix.

The GCRMA estimated TagD log2 expression ratio is 1.027 with an NLP of 2.90. The lower NLP relative to RMA might result from the subtraction of "pseudo-MM" intensities, derived from the nucleotide sequences of PM probes. In fact, GCRMA associates 27 false positives and 48 true positives with this NLP. The corresponding FDR is 36% and the highest false-positive log2 expression ratio is below 0.4. This GCRMA result is conditionally adequate for reliable detection of spiked differential expression when all samples share the same background hybridization mix.

In this example, the performance of RMA and GCRMA is significantly better than MAS5 while the performance of WPP is by far the best.

Example 2: Accuracy and reliability of estimated spike expression ratios for experiments 7 and 8

The table below presents descriptive statistics for NLP, L2ER (Log2 Expression Ratio) and FP (False Positives) based on the sets of forty 2:1 spikes that have the highest NLP values for each algorithm. The false positives for each algorithm consist of the non-spikes that have NLP values lying within the range defined by the corresponding set of forty spikes.

	MAS5	RMA	GCRMA	WPP
NLP				
max	5.663	6.679	6.817	15.346
mean	4.161	4.484	4.825	14.426
median	4.180	4.276	4.813	14.685
min	3.129	3.294	3.201	12.698
L2ER				
max	1.703	1.704	2.031	1.759
mean	1.111	0.983	1.376	1.049
median	1.071	1.007	1.407	0.992
min	0.612	0.392	0.770	0.497
FP				
count	42	15	15	0
FDR	51.22%	27.27%	27.27%	0.00%

The main features of this table confirm and/or extend features noted in Example 1:

The FDR (False Discovery Rate) is highest for MAS5, is significantly lower for both RMA and GCRMA and is zero for WPP (Supplementary figure 1).

The range of NLP values is exceptionally high for WPP while the relatively low ranges of the other NLP values increase steadily from MAS5 to RMA to GCRMA.

The L2ER (Log2 Expression Ratio) statistics for GCRMA reveal a strong shift toward values significantly above the correct value of 1, suggesting that the current version of GCRMA generally overcorrects for PM cross hybridization. No such bias appears in the WPP or MAS5 results.

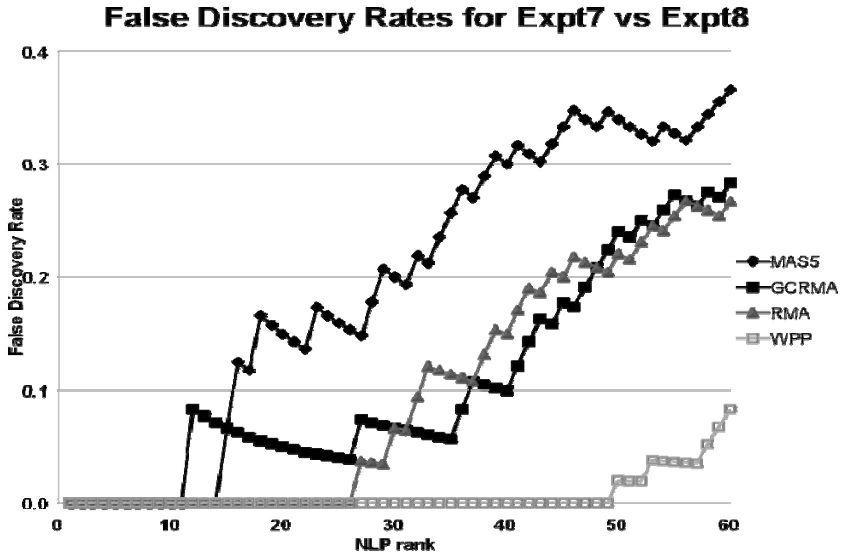

Supplementary figure 1: Dependence of the False Discovery Rate (FDR) on the rank of the cutoff NLP (Negative Log10 P-value) for detecting differential expression between Expt7 and Expt8 of the Affymetrix U133A Latin Square triplicate spike-in data. Each FDR value equals the proportion of false detections for the corresponding cutoff NLP value. All statistical tests were performed on all probe sets. P-values for MAS5, RMA and GCRMA were calculated by applying parametric t-tests to the two sets of triplicate expression estimates. P-values for WPP were calculated by applying non-parametric Mood tests to the two sets of bias-corrected PM probe intensities.

Example 3: Correction of bias caused by uninformative PM probes

This example shows how WPP solves a problem that apparently affects up to 90% of all probesets and arises because up to 40% of all PM probes are uninformative (Wendell Jones, “Are We Missing Obvious Differential Expression?” http://www.healthtech.com/2004/mda/day1.asp).

Only 5 of the 11 PM probes in probeset 205397_x_at are informative (i.e. sensitive to the spiked SMAD3 transcript), while a majority of the PM probes are uninformative (i.e. show little or no sensitivity to the transcript). Consequently the median PM probe intensity is completely determined by uninformative probes and is itself uninformative.

WPP iteratively excludes 5 of the 6 uninformative probes, causing the median of the included PM probe intensities to become informative. In fact, <u>the maximum WPP estimated expression ratio exceeds 100:1 and is not exceeded by any non-spiked WPP</u>

expression ratio. This result is reasonably accurate and is more than adequate for reliable detection of spiked differential expression.

MAS5 applies a Tukey-biweight procedure to reduce the weights of outlier intensities which lie far from the median. Consequently, the weights of the 5 sensitive PM probes are significantly reduced. In fact, the maximum MAS5 estimated expression ratio is less than 12:1 and is exceeded by 43 non-spiked MAS5 estimated expression ratios. This result is highly inaccurate and is also inadequate for reliable detection of spiked differential expression, even when all samples share the same background hybridization mix.

RMA employs a median polish procedure to fit an additive model of log probe intensities, ignoring outliers. Consequently, the 5 sensitive PM probes have little or no influence on the final values of the fitted model parameters. In fact, the maximum RMA estimated expression ratio is less than 2:1, but it is not exceeded by any non-spiked RMA estimated expression ratios. This result is highly inaccurate, but it is still conditionally adequate for reliable detection of spiked differential expression when all samples share the same background hybridization mix.

GCRMA precedes median polish with a theoretical sequence-specific correction for PM cross hybridization, which tends to increase the GCRMA estimated expression ratios (cf. Example 2). In this example, the maximum GCRMA estimated expression ratio is almost 6:1 and remains higher than any non-spiked GCRMA estimated expression ratios. This GCRMA result is less inaccurate than the RMA result and is also conditionally adequate for reliable detection of spiked differential expression when all samples share the same background hybridization mix.

As in the other examples, the performance of RMA and GCRMA is significantly better than MAS5 while the performance of WPP is by far the best.
